# The Utility of Chest Imaging for Surveillance of Atypical Lipomatous Tumors

**DOI:** 10.1155/2021/4740924

**Published:** 2021-10-11

**Authors:** Alexander L. Lazarides, Harrison R. Ferlauto, Zachary D. C. Burke, Anthony M. Griffin, Bruce D. Leckey, Nicholas M. Bernthal, Jay S. Wunder, Peter C. Ferguson, Julia D. Visgauss, Brian E. Brigman, William C. Eward

**Affiliations:** ^1^Department of Orthopaedic Surgery, Duke University Medical Center, Durham, NC, USA; ^2^Department of Orthopedic Surgery, University of California Los Angeles Hospital, Los Angeles, CA, USA; ^3^Department of Orthopedic Surgery, Mt Sinai Hospital, Toronto, Canada; ^4^Department of Pathology, Duke University Medical Center, Durham, NC, USA

## Abstract

**Background:**

Unlike other soft tissue sarcomas, atypical lipomatous tumors (ALTs) are thought to have a low propensity for metastasis. Despite this, a standard of care for pulmonary metastasis (PM) surveillance has not been established. This study aimed to evaluate the utility of chest imaging for PM surveillance following ALT excision.

**Methods:**

This was a multi-institution, retrospective review of all patients with primary ALTs of the extremities or superficial torso who underwent excision between 2006 and 2018. Minimum follow-up was two years. Long-term survival was evaluated using the Kaplan–Meier method.

**Results:**

190 patients with ALT were included. Average age was 61.7 years and average follow-up was 58.6 months (24 to 180 months). MDM2 testing was positive in 88 patients (46.3%), and 102 (53.7%) did not receive MDM2 testing. 188 patients (98.9%) had marginal excision, and 127 (66.8%) had marginal or positive margins. Patients received an average of 0.9 CT scans and 1.3 chest radiographs over the surveillance period. 10-year metastasis-free survival was 100%, with no documented deaths from disease.

**Conclusions:**

This study suggests that chest imaging does not have a significant role in PM surveillance following ALT excision, but advanced local imaging and chest surveillance may be considered in cases of local recurrence or concern for dedifferentiation.

## 1. Introduction

Adipocytic neoplasms comprise a spectrum of soft tissue tumors ranging from benign lipomas to high-grade liposarcomas [1]. Within this spectrum exists a low-grade adipocytic neoplasm that, prior to 1979, was known as a well differentiated liposarcoma (WDL) [2]. The clinical course of this low-grade soft tissue sarcoma (STS) is highly dependent on its anatomic location of origin [3–6]. In particular, WDLs arising in the retroperitoneum tend to dedifferentiate, recur, and metastasize more frequently than WDLs arising in the extremities or superficial torso. As such, despite being genetically, histologically, and grossly identical to WDLs of the retroperitoneum, WDLs of the extremities and superficial torso are now referred to as atypical lipomatous tumors (ALTs). This change in nomenclature reflects the fact that these tumors behave less like malignant liposarcomas and more like locally aggressive, benign lipomas [7].

ALTs account for approximately 25% of all deep lipomatous tumors of the extremities and are most frequently located in the thigh [8]. Historically, ALTs were difficult to distinguish from benign lipomas as the two lesions have an overlapping gross, histologic, and radiologic appearance [9, 10]. However, the recent identification of amplification of the MDM2 oncogene in ALTs has facilitated accurate differentiation from benign lipomas. Testing for this gene amplification via fluorescence *in situ* hybridization (FISH) has dramatically improved diagnostic accuracy, with a sensitivity and specificity for ALT now approaching 100% [11, 12]. Surgical excision is the mainstay of treatment for ALTs for which resection is chosen, but there is debate as to whether wide or marginal excision should be performed. Although wide excision appears to be associated with a slightly lower rate of local recurrence, recent analyses have demonstrated acceptable outcomes after marginal excision, with local recurrence occurring approximately 15% of the time [13, 14]. Despite a propensity for local recurrence, metastasis is extremely rare and is thought to occur in <1% of cases [13].

The utility of chest imaging for surveillance of pulmonary metastasis (PM) in STS of the extremities is well established [15, 16]. However, the utility of chest imaging for PM surveillance in ALTs is not well defined given their low metastatic potential. Current National Comprehensive Cancer Network (NCCN) guidelines only provide general recommendations for STS surveillance, based primarily on disease stage, and are limited by a lack of literature on effective surveillance strategies [17]. Due to this paucity of specific guidelines, there is currently significant interclinician variability in surveillance strategies, especially for low-grade lesions such as ALTs [18–20]. In practice, there are a variety of chosen surveillance practices, many of which include periodic chest imaging. A recently proposed follow-up schedule for surveillance of extremity STS did offer more specific, evidence-based recommendations for STS surveillance, but did not explicitly provide guidance on ALTs [21]. Thus, there exists a need for a specific, evidence-based strategy for PM surveillance in patients with ALTs. The purpose of this study was to evaluate the utility of chest imaging for surveillance of PM in patients who have had ALT excision.

## 2. Methods

In this multi-institutional cohort study, prospectively collected sarcoma databases from three tertiary referral sarcoma centers were reviewed retrospectively. Institutional review board approval was obtained prior to commencing study activities (Pro00101694). Included patients were those with a histopathologic diagnosis of ALT or WDL of an extremity or superficial torso, who subsequently underwent surgical excision between 2006 and 2018. These dates were selected to capture a modern cohort of patients both before and after the MDM2 era. A board-certified pathologist, experienced in sarcoma, confirmed the histopathologic diagnosis in each case. Margin status was assessed microscopically. For patients in whom MDM2 testing was performed, only patients with MDM2 positivity were included. Exclusion criteria were intrathoracic (i.e., pulmonary), abdominal, and retroperitoneal liposarcomas. Patients without histopathologic confirmation of ALT or WDL were also excluded, as were patients with less than two-year follow-up. It should be noted that all records were reviewed for presence of metastatic disease prior to exclusion. In patients without MDM2 testing, the diagnosis of ALT was made based on clinical and histopathological data as interpreted by a multidisciplinary group of experienced sarcoma surgeons, oncologists, and pathologists.

The primary outcome measure was PM. Metastatic disease was considered present if there was computed tomography (CT) scan showing evidence of pulmonary disease progression and a biopsy confirming metastasis. Secondary outcomes measures were cost of surveillance and local recurrence. Costs were calculated based on publicly available averages for CT scans of the thorax without contrast ($285) and chest radiographs with 2 views ($60.93) in North Carolina [22]. The typical manner of surveillance for local recurrence was physical exam, with advanced imaging performed in circumstances of clinical concern for recurrence. Local recurrence was considered present if there was MRI evidence of recurrence, or if there was a biopsy confirming recurrence.

Medical records were reviewed and patients meeting inclusion criteria were isolated. Demographic variables collected included age, sex, BMI, and follow-up time. Tumor and treatment characteristics collected included histopathologic diagnosis, MDM2 testing results (if available), type of surgical resection (wide vs. marginal), tumor location, size, and margin status. Variables relevant to our outcomes of interest included follow-up time, use of CT chest imaging from the time of diagnosis, use of chest radiographs from the time of diagnosis, office visits, evidence of metastasis, evidence of local recurrence, and both overall and disease-free survival time. We also noted any incidental findings of surveillance chest imaging that prompted further intervention. Marginal resections were considered as having positive margins, unless noted otherwise.

Categorical data were expressed as whole numbers, and continuous data were expressed as means and ranges. Univariate analyses were used to identify differences in baseline characteristics between patients with and without MDM2 testing. Long-term survival was evaluated using the Kaplan–Meier (KM) method. JMP Pro 15 (SAS Inc, Cary, NC) was used for all statistical analyses, with *p* < 0.05 denoting significance.

## 3. Results

### 3.1. Patient Characteristics

Our initial review identified 285 patients with a diagnosis of ALT. Seventy-nine patients were excluded for having less than two-year follow-up. Sixteen patients were excluded for having a negative MDM2 test. This left 190 patients with a diagnosis of ALT who were included for analysis (Table 1). The average age was 61.7 years, and the average follow-up was 58.6 months (range, 24 to 180 months). One hundred patients (52.6%) were male. The average BMI was 28.1 (range, 16.3 to 50.3). MDM2 testing of the tumor was positive in 88 patients (46.3%), and 102 patients (53.7%) did not have MDM2 testing performed. The average tumor size was 17.7 cm (range, 1.1 to 45.7 cm) in greatest dimension. One hundred fifty-six tumors (82.1%) were located in the lower extremity, 25 (13.2%) in the upper extremity, and nine (4.7%) in the superficial torso. All patients included underwent surgical resection; nearly all of patients (188 patients, 98.9%) received either a marginal or intralesional excision; final pathology revealed either macroscopically or microscopically positive margins in 127 patients (66.8%). The remaining 49 patients had negative margins while 19 patients had a margin status that was not determinable owing to the well-differentiated nature of the disease and inability to differentiate from the normal surrounding tissue.

**Figure 1:**
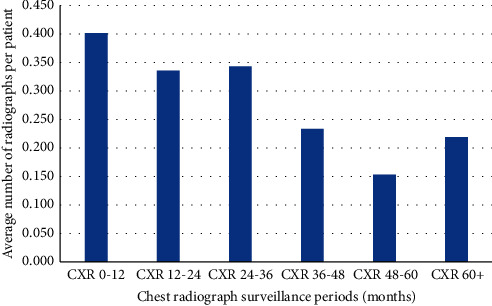
Histogram demonstrating the distribution of chest radiograph follow-up averaged per patient. The average imaging follow-up of patients undergoing chest radiograph follow-up was 50 months.

**Figure 2:**
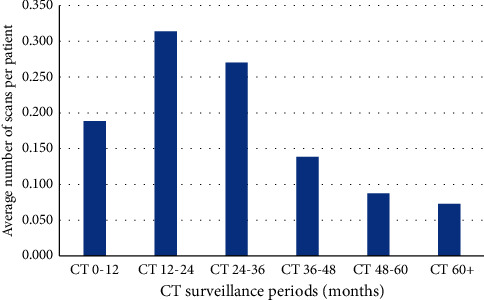
Histogram demonstrating the distribution of computed tomography (CT) follow-up averaged per patient. The average imaging follow-up of patients undergoing CT imaging follow-up was 47.6 months.

Patients received an average of 1.3 chest radiographs (range, 0 to 14) and average of 0.9 CT scans (range, 0 to 11) over the surveillance period. There was heterogeneity in the number and manner of chest surveillance performed over the study period. The weighted distribution of when and how many of these were performed over the surveillance period is displayed for chest radiographs and CT scans in Figures 1 and 2, respectively. Based on US average cost data, the average cost of chest surveillance imaging was $323.58 (range, $0 to $2678.40). From 161 CT scans of the chest, the number of incidental findings prompting further intervention was two, consisting of two symptomatic hiatal hernias.

When investigating patients with and without MDM2 testing, the study cohorts were similar. Patients with MDM2 testing tended to have larger tumors (19.3 vs. 16.3 cm, *p* < 0.02) and shorter follow-up time (47.2 vs. 68.3 months, *p* < 0.001). There was no difference in rates of local recurrence or metastasis between the groups.

### 3.2. Metastasis- and Recurrence-Free Survival

There was a single lesion concerning for PM by chest CT that occurred after a patient had a local recurrence. This individual's primary tumor did not receive MDM2 testing, and the histopathologic diagnosis was ALT, sclerosis type. The patient had a recurrence with rapid growth and imaging characteristics concerning for dedifferentiation; however, this was not biopsied, and the patient declined further management. The patient then presented with acute respiratory failure and was found to have a pleural effusion and pleural carcinomatosis. She also declined biopsy of the lung lesions but did consent to thoracentesis. This showed cells with MDM2 rare positivity but was also positive for WT-1, Keratin, and S100, which was deemed by pathology to be nonspecific. The patient died of a myocardial infarction before the lesion could be biopsied and proven to be PM. Excluding this case, the 10-year metastasis-free survival was 100%. No patient had documented death from disease (Figure 1).

**Figure 3:**
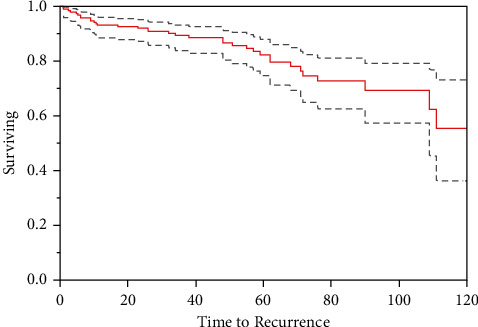
10-year recurrence-free survival with 95% confidence intervals represented by dotted lines.

Twenty-five patients (13.2%) had a local recurrence within five years of surgery (Figure 2). An additional 11 patients (5.8%) had a local recurrence between five and ten years (Figure 3). Thus, 36 patients in total (18.9%) had a local recurrence within 10 years. Of these patients, 16 (44.4%) were positive for MDM2 while 20 (55.6%) did not have MDM2 testing performed. A single local recurrence later recurred again as a biopsy-proven high-grade liposarcoma. This tumor was MDM2 positive and was located in the anterior arm. The original resection was margin positive. This patient ultimately underwent a shoulder disarticulation. At most recent follow-up (12 months), the patient remained negative for metastatic disease.

## 4. Discussion

For STS of the extremities and for WDL of the retroperitoneum, chest surveillance after surgical resection is a critical component of management. However, for ALTs, the role of PM surveillance is less clear. While local recurrence is not infrequent, metastases are rare. The goal of this study was to investigate the utility of chest imaging in the surveillance of ALTs. We found that, for ALTs without local recurrence, chest surveillance does not appear to alter oncologic outcomes and instead appears to contribute an unnecessary added cost.

One may question whether there are any downsides to intensive chest surveillance for low-grade sarcomas. Recent analysis has demonstrated that such a strategy may have significant fiscal and emotional costs to patients. A systematic review by Goel et al. found that costs related to chest surveillance have wide discrepancies based on grade and surveillance strategy and that costs for follow-up of low-grade tumors average $485 [23]. These findings are consistent with those of our study, which found that the average cost of chest surveillance was approximately $323. Unnecessary surveillance has also been shown to place a significant burden on the workflow of the sarcoma clinic [24]. More important, though, are considerations of the impact on the patient. Findings of a pulmonary nodule of unknown significance can cause patients significant emotional distress [25]. In addition, studies of chest surveillance for lung cancer have suggested an increased risk of carcinogenesis secondary to the radiation exposure of surveillance chest imaging [26]. In all, these findings suggest that unnecessary surveillance should be avoided if the costs outweigh the benefits.

While there is agreement that surveillance is important for sarcoma management as a whole, there is great discrepancy in actual practice. Gerrand et al. surveyed 155 clinicians and found wide variations in modalities and intervals utilized for sarcoma surveillance [19]. A study by Puri et al. provided high level evidence to help guide clinicians in selecting the modality and interval of chest surveillance [27]. In that study, 476 patients who underwent resection of an extremity sarcoma were enrolled to compare surveillance with chest radiographs versus CT scans, as well as to compare surveillance with a less intensive follow-up protocol (every six months) versus a more intensive one (every three months). They found that chest radiographs—despite being inherently less sensitive for detecting PM than CT scans—were sufficient to detect most pulmonary metastases without deleterious effects on the eventual outcome and that survival was noninferior in the less intensive surveillance group. The current study supports these findings for ALTs, as surveillance chest CT scans offered no added benefit over chest radiographs for surveillance in this patient cohort. Thus, our study suggests that should physicians deem chest surveillance to be necessary, chest radiographs are likely sufficient.

In our study, there was significant heterogeneity in surveillance patterns. As reflected by the average number of radiographs and scans, some surgeons employed a more rigorous and intensive follow-up schedule while others did not routinely perform surveillance unless other concerning features, such as local recurrence or specific tumor characteristics, prompted otherwise. While each patient's tumor should be managed on an individual basis, the general suggestion of the present study is that a less rigorous chest surveillance protocol did not result in worse survival for the patients.

Developing a rational protocol for surveillance requires an estimation of the annual event rate of metastasis. Wilson et al. developed such an evidence-based follow-up schedule for extremity sarcomas [21]. They found that, for low-grade sarcomas, annual surveillance for 5 years is likely sufficient. Their study was a step in the right direction and made delineations based on grade and size. However, their study made no specific recommendations for ALTs, which are unlike many other sarcomas with respect to biology and propensity for metastasis. Studies on long-term survival of ALTs are limited. Lazarides et al. investigated the utility of radiotherapy in 1418 patients with WDL [28]. They found that surgery alone provided a >90% 5-year survival rate. While this study provided insight that neither margin status nor adjuvant therapies appeared to affect survival, it lacked granular details regarding tumor characteristics and metastasis-free survival. A study by Fisher et al. found that, among 63 patients with ALTs, the metastasis rate was 0% [29]. While a small cohort, these findings are consistent with the findings in this study. Indeed, in our study, the annual event rate for metastasis of primary ALTs was zero, suggesting that the role of chest surveillance is limited.

One patient included in this study presented in follow-up with clinical concern for dedifferentiation of a previously diagnosed ALT, as well as concern for PM. This patient was not included as a metastasis in our analysis as the predetermined criteria of biopsy-proven PM were not met. If the pulmonary disease observed in this patient did, in fact, represent metastatic lesions, it is important to note that imaging of the primary tumor was highly concerning for dedifferentiation. Thus, it may be reasonable to extrapolate that while the findings in this study suggest that chest surveillance may be overutilized in primary ALTs, clinicians should assess the need for PM surveillance in the setting of clinical concern for dedifferentiation. It is our practice to perform an image-guided biopsy of any portions of a fatty tumor which on imaging show concern for dedifferentiation (i.e., areas that have signal characteristics on MR imaging which differ from fat). Pertinent to this discussion, it is important to note that this study excluded all patients with confirmed evidence of dedifferentiation at initial surgical resection. Of the patients with an initial confirmed ALT without dedifferentiation, only 1 (0.5%) of the patients ultimately developed a recurrence with a biopsy-proven high-grade or dedifferentiated component. This is germane to the consideration of chest surveillance patterns, as these patients would certainly be at higher risk for the development of PMs; our study lends credence to the idea that the rate of dedifferentiation after initial resection of an isolated ALT is actually very low. Given such a low rate of dedifferentiation, consideration of routine chest CT imaging becomes even less attractive from a cost-benefit perspective.

This study is not without limitations. First, this is a retrospective cohort study. As such, surveillance patterns were not dictated in a uniform manner and were often decided upon by the patient and surgeon. In taking this into account, it is important to note that we are unable to definitively comment as to the status of the lungs at final follow-up, as would be confirmed by chest CT imaging. While an early surveillance CT may be negative for PMs, this may not predict that it will be always negative for ALTs; more typically, chest radiographs were utilized for surveillance, and these may lack the sensitivity for PMs of a CT scan. Despite this, we consider that an important conclusion from this study, though, is that, even with limited surveillance imaging data, confirmed death from disease was rare. Second, many patients were excluded from this study owing to insufficient follow-up. It is possible that including this cohort of patients could have altered our findings; however, we feel that including only patients with minimum 2-year follow-up provided a more rigorous and conservative estimation of the metastasis rate of ALTs. Postoperative follow-up duration, which was 5 years on average, might also be too short to evaluate metastatic potential of a low-grade tumor such as an ALT. As such, it is possible that late metastases would not be detected in our study design. It also warrants noting that approximately half of our patients lacked MDM2 testing. These patients were diagnosed with ALT by a multidisciplinary sarcoma team of surgeons, medical oncologists, and pathologists after review of histopathological and clinical data. It is possible that we included patients who did not in fact have an ALT and instead had a lipoma. Despite this, there were not significant differences in outcomes between the cohort of patients with MDM2 positive testing and the cohort with unknown MDM2 status, suggesting the two groups may be similar. Furthermore, these data may be relevant to surgeons treating low-grade lipomatous tumors in the absence of MDM testing, suggesting that similar surveillance strategies may be used in tumors that are deemed to be ALT by clinical and histopathological data alone. Finally, although the primary aim of this study was to investigate the utility of chest imaging, we did observe a significant rate of local recurrence (18.9% at 10 years). Thus, we do believe there is utility in surveilling patients for local recurrence and that patients with local recurrence should be more closely followed up for a subsequent PM; however, this warrants further investigation. Overall, these limitations could be improved with a standardized regimen for diagnosis and surveillance, validated in a prospective, longitudinal manner.

## 5. Conclusion

For ALTs, the utility of chest imaging for surveillance of PM has not been well defined. Within this multi-institution cohort of 190 patients, we found no cases of PM despite a wide variety of types and durations of chest surveillance. This suggests that chest imaging does not have a significant role in the surveillance of ALTs without local recurrence. As such, we suggest that surveillance should focus on detecting local recurrence. Advanced local imaging and chest surveillance may be considered in cases of local recurrence or concern for dedifferentiation.

## Figures and Tables

**Table 1:** Baseline demographic and tumor characteristics.

Variable	Overall (*n* = 190)		MDM2 positive (*n* = 88)		MDM2 not performed (*n* = 102)		*p* value
	*n*	(%)	*n*	(%)	*n*	(%)	
Age (mean, yrs)^*∗*^	61.7 ± 13.1		62.2 ± 13.7		61.4 ± 12.6		0.67
Sex (male)	100	52.6	46	52.3	54	52.9	0.93
BMI (mean)^*∗*^	28.1 ± 5.2		28.4 ± 5.3		27.8 ± 5.1		0.44
Margin status							0.22
Positive	127	66.8	63	71.6	64	62.7	
Negative	49	25.9	16	18.2	33	37.3	
Could not be determined	14	7.4	9	10.2	5	4.9	
Location							0.98
Upper extremity	25	13.2	12	13.6	13	12.7	
Lower extremity	156	82.1	73	83.0	83	81.4	
Superficial torso	9	4.7	3	3.4	6	5.9	
Size (cm)^*∗*^	17.7 ± 8.5		19.3 ± 7.8		16.3 ± 9.0		**0.02**
CT scans (mean)^*∗*^	0.9 ± 2.1		0.7 ± 2.6		1 ± 1.7		0.39
CXRs (mean)^*∗*^	1.3 ± 2.5		1.4 ± 2.3		1.3 ± 2.8		0.61
Local recurrences (total)	36	18.9	16	18.2	20	19.6	0.75
Cost (mean, $)^*∗*^,^^^	323.58 ± 585.95		371.40 ± 689.57		281.08 ± 475.01		0.25
Follow-up time (mean, months)^*∗*^	58.6 ± 33.1		47.2 ± 27.7		68.4 ± 34.2		**<0.001**

^*∗*^Continuous variables presented with mean and SD. ^Costs were calculated based on publicly available averages for CT scans of the thorax without contrast ($285) and chest radiographs with 2 views ($60.93) in North Carolina. Bold values represent statistical significance at *p* < 0.05.

## Data Availability

The data that support the findings of this study are available on request from the corresponding author and are not publicly available due to privacy or ethical restrictions.
